# Critical features of acute stress-induced cross-sensitization identified through the hypothalamic-pituitary-adrenal axis output

**DOI:** 10.1038/srep31244

**Published:** 2016-08-11

**Authors:** Xavier Belda, Roser Nadal, Antonio Armario

**Affiliations:** 1 Institut de Neurociències, Universitat Autònoma de Barcelona, Cerdanyola del Vallès 08193, Barcelona, Spain; 2 Animal Physiology Unit, School of Biosciences, Universitat Autònoma de Barcelona, Cerdanyola del Vallès 08193, Barcelona, Spain; 3 Psychobiology Unit, School of Psychology, Universitat Autònoma de Barcelona, Cerdanyola del Vallès 08193, Barcelona, Spain; 4 Red de Trastornos Adictivos (ISCIII) Universitat Autònoma de Barcelona, Cerdanyola del Vallès 08193, Barcelona, Spain

## Abstract

Stress-induced sensitization represents a process whereby prior exposure to severe stressors leaves animals or humans in a hyper-responsive state to further stressors. Indeed, this phenomenon is assumed to be the basis of certain stress-associated pathologies, including post-traumatic stress disorder and psychosis. One biological system particularly prone to sensitization is the hypothalamic-pituitary-adrenal (HPA) axis, the prototypic stress system. It is well established that under certain conditions, prior exposure of animals to acute and chronic (triggering) stressors enhances HPA responses to novel (heterotypic) stressors on subsequent days (e.g. raised plasma ACTH and corticosterone levels). However, such changes remain somewhat controversial and thus, the present study aimed to identify the critical characteristics of the triggering and challenging stressors that affect acute stress-induced HPA cross-sensitization in adult rats. We found that HPA cross-sensitization is markedly influenced by the intensity of the triggering stressor, whereas the length of exposure mainly affects its persistence. Importantly, HPA sensitization is more evident with mild than strong challenging stressors, and it may remain unnoticed if exposure to the challenging stressor is prolonged beyond 15 min. We speculate that heterotypic HPA sensitization might have developed to optimize biologically adaptive responses to further brief stressors.

Animals exposed to acute stress immediately respond by altering their behavior and physiology. Typical behavioral changes involve reduced activity and enhanced anxiety, particularly in response to relatively severe stressors^1^,^2^. Prototypic physiological changes include the activation of components of the autonomous nervous system and the hypothalamic-pituitary-adrenal (HPA) axis. The activation of the former mainly affects its sympathetic branch, provoking important cardiovascular changes, and the release of noradrenaline and adrenaline into the bloodstream. Stress-induced activation of the HPA axis results from a convergence of the inputs to the medial parvocellular subdivision of the hypothalamic paraventricular nucleus (mpdPVN), which contains the neurons that synthesize the corticotropin-releasing hormone (CRH) and other ACTH secretagogues (e.g. vasopressin)^3^. CRH is considered the main hypothalamic stimulatory factor that controls both the synthesis and release of ACTH by corticotropin cells of the anterior pituitary. In turn, ACTH controls the synthesis and secretion of glucocorticoids from the adrenal cortex: cortisol in humans and most mammals; corticosterone in rats and mice. Stress-induced glucocorticoid release has a wide range of central and peripheral effects, including the mobilization of resources, the modulation of immune function and negative feedback^4^,^5^.

Although acute stress-induced changes are likely to be transient after exposure to mild stressors, exposure to severe and uncontrollable stressors can leave a trace that lasts for several days or weeks, and that may be reflected in neurochemical, neuroendocrine and behavioral changes^2^,^6^,^7^. Indeed, the long-lasting behavioral effects of severe stressors are reminiscent of behavioral alterations observed in humans exposed to traumatic experiences. As such, prominent behavioral perturbations are initially observed that vanish over days or weeks in most subjects, while in particularly susceptible individuals these perturbations persist for longer and they can lead to post-traumatic stress disorders and/or depression^8^.

Sensitization is an important consequence of exposure to certain stressors, a state of hyper-responsiveness to other stressful situations. Sensitization is particularly relevant to physiological pathologies like irritable bowel disease or chronic pain, as well as for psychiatric conditions such as post-traumatic stress disorder (PTSD), schizophrenia and drug addiction^9^,^10^,^11^,^12^,^13^. In animals, it is well-known that a prior history of acute stress can sensitize certain behavioral and physiological responses to novel, subsequent (heterotypic) stressors^2^,^6^,^14^, and this has been particularly well-characterized with regard to the HPA axis^7^. However, there are many cases in which prior exposure to an acute stressor does not alter the response to further stressors when typically tested in the following 24 h^15^. It is note that in terms of the HPA axis, cross-sensitization is typically referred to as facilitation^16^, yet here we prefer to use the more general concept of cross-sensitization or heterotypic sensitization.

Unfortunately, to date the characteristics that determine the magnitude and duration of stress-induced heterotypic HPA sensitization have not been studied, and several other critical questions remain unanswered. The severity of the triggering stressor is likely to have a major contribution, since HPA sensitization has mainly been reported after exposure to immobilization on boards (IMO) or electric tail-shock protocols that are typical of the classical learned helplessness procedure^17^,^18^,^19^,^20^,^21^,. Stressors may differ greatly and they are often difficult to compare^22^. Indeed, they appear to differ in terms of intensity, a property that can be objectively evaluated using classical biological stress markers like plasma catecholamines, HPA hormones and prolactin^23^. We are unaware of any studies that have used these biological markers to directly compare the tail-shock procedure with other stressors, yet IMO appears to be consistently stronger in our hands than other stressors like restraint, forced swim or foot-shock^24^,^25^,^26^,^27^,^28^. It seems reasonable to assume that the intensity of stressors will be an important influence on HPA cross-sensitization, even though this hypothesis remains to be tested. In addition, there is considerable evidence that acute stress-induced HPA sensitization is not only dependent on the intensity and/or length of the triggering stressor but also, on the duration of the heterotypic stressor. Accordingly, such effects are better observed after short-exposure to the challenging stressor^17^,^18^. This is important as heterotypic HPA sensitization could go unnoticed in sub-optimal conditions.

Therefore, this study aimed to specifically assess whether acute stress-induced HPA cross-sensitization is only observed with severe triggering stressors, and if more prolonged exposure to the acute stress could potentiate HPA sensitization. Moreover, we tested whether the length of exposure to the stressor might be critical to detect HPA sensitization (see Table 1 for a summary of the experimental design).

## Results

### The intensity of the triggering stress influences cross-sensitization (Experiment 1)

To demonstrate whether the intensity of the initial TRIGGERING STRESSOR affects cross-sensitization of the HPA axis and hence, its response to novel subsequent stressors, animals were assigned to four groups on day 1 (10 rats per group): CONTROL, maintained in their home cages; SWIM, subjected to forced swim for 30 min; SHOCK, receiving 30 intermittent foot-shocks over 30 min; and IMO, subjected to IMO for 30 min. These stressors were chosen on the basis that they differ in terms of intensity, as evaluated using various stress markers^27^,^28^. On day 2, all the animals were introduced individually into an open field (OF) for 15 min and their blood was sampled immediately after the test.

**Figure 1:**
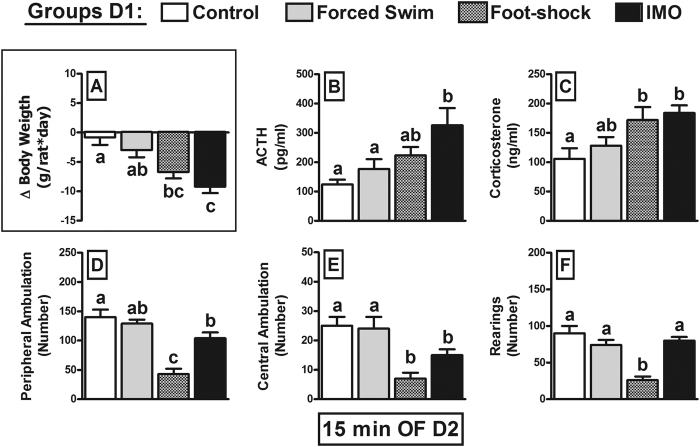
The influence of prior exposure to stressors (30 min) on the HPA and the behavioral response to an open-field test (15 min) on the following day. The means and SEM (n = 10 per group) are shown and the groups labelled with different letters differ statistically (p < 0.05). The intensity of the triggering stressors (forced swim, foot-shock, IMO) was evaluated by the changes in body weight (**A**) from day 1 (D1) to day 2 (D2). The magnitude of ACTH and corticosterone cross-sensitization (**B**,**C**) roughly paralleled the body weight changes. Inhibition of OF activity (**D–F**) followed a different pattern such that prior foot-shock caused a stronger decrease than IMO.

The changes in body weight from day 1 to day 2 (Fig. 1A) were analyzed using a Generalized Linear Model (GzLM) and this showed a significant effect of the TRIGGERING STRESSOR (χ^2^(3) = 33.6; p < 0.001), whereby there was a significant reduction of body weight in the SHOCK and IMO rats, with the strongest effect observed after IMO. To evaluate cross-sensitization of the HPA axis, we measured the plasma ACTH (Fig. 1B) and corticosterone (Fig. 1C) after a 15 min exposure to the OF, revealing a clear effect of the TRIGGERING STRESSOR on both ACTH (χ^2^(3) = 17.4; p = 0.001) and corticosterone (χ^2^(3) = 14.9; p = 0.002). *Post-hoc* comparisons of the ACTH levels revealed that prior foot-shock tended to enhance the response to the OF, yet such enhancement was only significant after prior IMO. By contrast, prior foot-shock and IMO increased corticosterone levels in response to the OF to the same extent.

An analysis of the behavior in the OF highlighted a significant effect of the TRIGGERING STRESSOR on peripheral ambulation (Fig. 1D; χ^2^(3) = 63.1; p < 0.001), central ambulation (Fig. 1E; χ^2^(3) = 27.0; p < 0.001) and rearing (Fig. 1F; χ^2^(3) = 57.0; p < 0.001). Whereas the rats in the prior SWIM group were not affected, and prior IMO reduced both peripheral and central ambulation but not rearing, the SHOCK groups displayed the strongest impact of the stressor, which markedly dampened all behaviors.

### Cross-sensitization is also affected by the challenging stressor (Experiment 2)

We tested how the characteristics of the novel CHALLENGING STRESSOR affect HPA cross-sensitization, both intensity and duration. On day 1, animals were either left undisturbed (CONTROL, n = 26) or they were exposed to IMO for 2 h (IMO, n = 26). On day 2, the rats in each group were exposed to either an OF for 5 min (n = 9), or to forced swim for 5 (n = 9) or 30 min (n = 8). These two times were chosen to study the influence of the duration of the challenging stressor comparing the response to a 5 min exposure with that typically used in most experiments studying cross-sensitization (30 min)^29^,^30^. Blood samples were taken immediately after the period of stress (END) and again 30 min later (R30). This latter time point was added as sampling immediately after a 5 min stress may not accurately assess the corticosterone response but only reliably measure the ACTH changes. The statistical analysis of the effects of the different CHALLENGING STRESSORS on day 2 was performed separately as the differences in stress exposure time did not allow appropriate comparisons between the three conditions.

**Figure 2:**
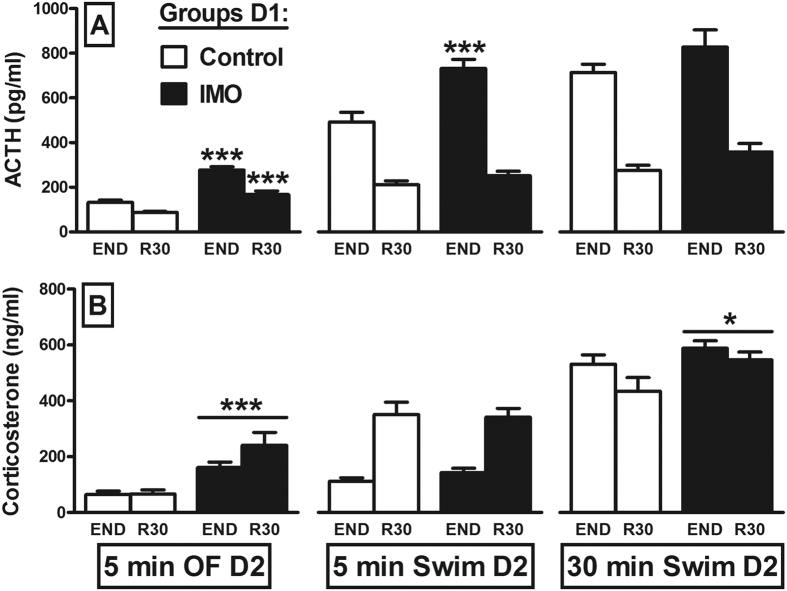
IMO-induced HPA cross-sensitization is dependent on the characteristics of the challenging stressors. The means and SEM (n = 8–9 per group) of ACTH and corticosterone plasma levels are shown. The rats exposed to 2 h IMO on day 1 (D1) were exposed to a 5 min open-field (OF) or to 5 or 30 min forced swim on the following day (D2), and their blood was sampled just after the stressors (END) and again 30 min later (R30): *p < 0.05, ***p < 0.001 relative to the respective sampling time in the control group.

A Generalized Estimating Equations (GEE) analysis of the ACTH response to the 5 min OF (Fig. 2A) highlighted a significant effect of PRIOR IMO (χ^2^(1) = 87.6; p < 0.001), TIME (χ^2^(1) = 53.2; p < 0.001) and their interaction (χ^2^ (1) = 9.6; p = 0.002). The *post-hoc* comparisons showed that prior exposure to IMO enhanced the ACTH response at both time points (p < 0.001 in both cases). In terms of the corticosterone response (Fig. 2B), a significant effect of PRIOR IMO alone was evident in the GEE analysis (χ^2^(1) = 21.7; p < 0.001), with a higher overall response in rats previously exposed to IMO.

GEE Analysis of the ACTH response to 5 min forced swim (Fig. 2A) highlighted a significant effect of PRIOR IMO (χ^2^(1) = 15.5; p < 0.001), TIME (χ^2^(1) = 212.6; p < 0.001) and their interaction (χ^2^(1) = 14.2; p < 0.001). Prior exposure to IMO only enhanced the ACTH response immediately after swim (END; p < 0.001). By contrast, the GEE analysis showed only a significant effect of TIME on corticosterone (Wald χ^2^(1) = 21.7; p < 0.001: Fig. 2B), with no evidence of cross-sensitization. Finally, the GEE analysis of the ACTH response to 30 min swim (Fig. 2A) showed no effect of PRIOR IMO but a significant effect of TIME (χ^2^(1) = 250.6; p < 0.001). In terms of the corticosterone response (Fig. 2B), the GEE analysis showed a significant effect of PRIOR IMO (χ^2^(1) = 4.7; p = 0.031) and TIME (Wald χ^2^(1) = 7.5; p = 0.006), indicating mild cross-sensitization.

### The influence of the duration of stress on cross-sensitization (Experiment 3)

In addition to the severity of the triggering stressors, the LENGTH of exposure to them might also influence the induction of HPA cross-sensitization. Thus, we studied the influence of the duration of the initial triggering stressor and we also compared the effect of prior prolonged exposure to IMO with that of an exogenous corticosterone administration schedule that mimicked the release of corticosterone during prolonged IMO. On day 1, the rats remained undisturbed (CONTROL, n = 10) or they were subjected to 5 min, 30 min or 4 h of IMO (n = 8 per group). An additional group of rats was administered corticosterone (5 mg/kg) twice (Sigma-Aldrich, Barcelona, Spain), at time 0 and 2 h later (B[5+5]; n = 8). Blood samples were collected from the CONTROL and IMO groups following the same schedule: just after IMO and again 45 min later (R45) with an additional sample taken from the 4 h IMO group at 1 h of IMO. Blood samples were collected from the B[5+5] animals at 1, 4 and 4:45 h after the first administration of corticosterone in order to compare the plasma ACTH and corticosterone levels with those in the 4 h IMO group. On day 2, all animals were exposed to 5 min OF (the optimum conditions to detect cross-sensitization) and blood was sampled immediately after the stress (END) and at R30. While OF behavior was videotaped, only the first 4 minutes of behavior were analyzed because for 4 rats the video camera inadvertently stopped within 5 min.

**Figure 3:**
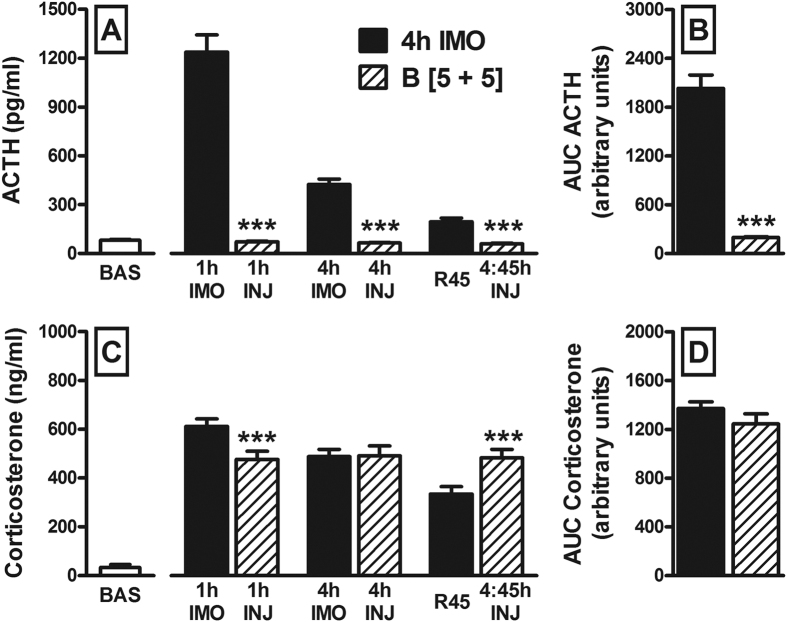
Comparison of the ACTH and corticosterone plasma levels after exposure to IMO for 4 h or to exogenous corticosterone administration (B [5+5]). The means and SEM are shown. Panels A and C show the resting levels in the CONTROL group (BAS; n = 10) and the dynamics of the hormonal responses to the treatments (n = 8 per group). Blood samples were taken from the two groups following the same schedule: at 1 h IMO, just after IMO (4 h IMO) and again 45 min later (R45) in the 4 h IMO group; and at 1, 4 and 4:45 h after the first administration of corticosterone in the B [5+5] group. The areas under the curve (AUC) of the hormonal responses are shown in panels B and D: ***p < 0.001 versus 4 h IMO group.

We compared the ACTH response to 4 h IMO and B[5+5] on day 1 (Fig. 3A) and the GEE analysis showed a significant effect of TREATMENT (χ^2^(1) = 140.7; p < 0.001), TIME (χ^2^(2) = 136.8; p < 0.001) and their interaction (χ^2^(2) = 131.4; p < 0.001). *Post-hoc* comparisons showed that 4 h IMO provoked higher levels of ACTH at all the time points studied (p < 0.001 in all cases). Consistent with these data, the Student’s *t* test revealed that the area under the curve (AUC) of the ACTH response was also higher in the 4 h IMO rats (p < 0.001: Fig. 3B). The GEE analysis of the corticosterone response (Fig. 3C) showed no effect of TREATMENT (χ^2^(1) = 0.02; ns) but a significant effect of TIME (χ^2^(2) = 46.2; p < 0.001) and the TREATMENT by TIME interaction (χ^2^(2) = 52.2; p < 0.001). Decomposition of the interaction showed that 4 h IMO group had more corticosterone at 1 h of IMO (p = 0.001), similar levels at 4 h of IMO and less at R45 (p < 0.001). When the AUCs of the corticosterone responses were compared (Fig. 3D) no TREATMENT effect was found (hormone data of the rest of the groups on day 1 can be seen in the Supplementary Table 1).

**Figure 4:**
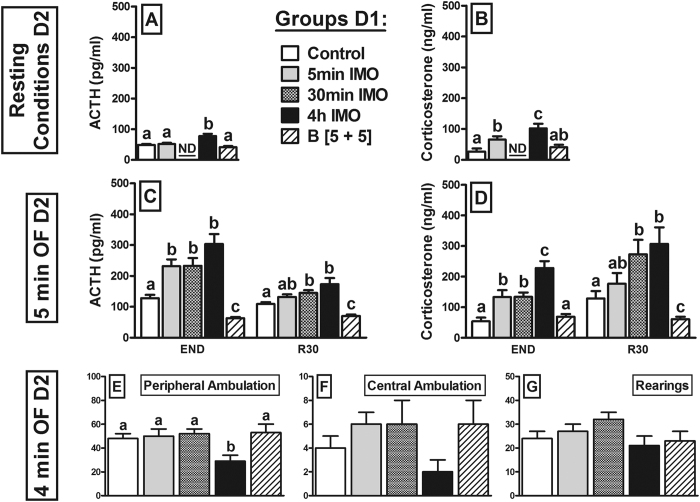
Prior exposure to different periods of IMO or to exogenous corticosterone administration (B [5+5]) on day 1 (D1) influences the HPA and the behavioral responses to a 5 min open-field test (OF) on the following day (D2). The means and SEM are shown and the groups labelled with different letters differ statistically (p < 0.05). Panels A and B show resting ACTH and corticosterone levels (n = 6 per group) from a different cohort of rats, and Panels C and D show the hormonal response to the OF (n = 8–10 per group). In the latter case, blood samples were taken just after the stressor (END) and again 30 min later (R30). Only 4 h IMO resulted in reduced OF activity (Panels E–G).

An additional cohort of rats was studied (excluding the 30 min IMO group) to determine how the treatments on day 1 affect the resting levels of ACTH and corticosterone on day 2 (n = 6 per group). The LENGTH of prior IMO exposure affected both the plasma ACTH (χ^2^(3) = 43.6; p < 0.001: Fig. 4A) and corticosterone (χ^2^(3) = 30.5; p < 0.001: Fig. 4B) levels and accordingly, the 4 h IMO group had higher resting levels of both hormones than the other groups. By contrast, exposure to 5 min IMO increased resting levels of corticosterone but not ACTH. Administration of exogenous corticosterone had no effect on the resting hormonal levels.

The GEE analysis of the ACTH response to 5 min OF on day 2 (Fig. 4C) highlighted a significant effect of LENGTH of prior IMO (χ^2^(4) = 298.5; p < 0.001), TIME (χ^2^(1) = 45.3; p < 0.001) and their interaction (χ^2^(4) = 54.1; p < 0.001). The ACTH response to the OF was enhanced in all the prior IMO groups, both at END and R30, and these effects were quite independent of the length of exposure to IMO. By contrast, prior corticosterone administration strongly inhibited the ACTH response. There was a significant effect of LENGTH of prior IMO (χ^2^(4) = 316.7; p < 0.001), TIME (χ^2^(1) = 15.1; p < 0.001) and their interaction on the corticosterone response ( χ^2^(4) = 38.0; p < 0.001: Fig. 4D). All prior IMO groups had higher levels of corticosterone at END, an effect that was more pronounced in the 4 h IMO group. However, while prior exposure to 30 min and 4 h IMO significantly increased the corticosterone response at R30, such an increase was not produced by 5 min IMO. Prior corticosterone administration did not affect the corticosterone response to the OF at END but it was profoundly inhibited at R30.

The statistical analysis of the impact of LENGTH of IMO on OF behavior showed a marked decrease on peripheral ambulation only after prior exposure to 4 h of IMO (χ^2^(3) = 15.9; p = 0.003: Fig. 4E). No significant effect was found on central ambulation or rearing activity (Fig. 4F,G, respectively).

### The effect of the duration of the triggering stress on the persistence of cross-sensitization (Experiment 4)

Although previous data suggest that the length of the triggering stressor influences the magnitude of HPA cross-sensitization, we wanted to assess if it also influences the PERSISTENCE of this phenomenon. Rats were subjected to IMO for 15 min or 2 h (n = 24), either 2 or 7 days (n = 6 per group) before being subjected to 10 min swim together with an additional control group (n = 6). One blood sample was obtained immediately after swim. Note that sub-optimal conditions of the heterotypic stressor were chosen to better detect group differences (swim instead of OF, and 10 min instead of 5 min). In this way we aimed to reduce the probability of finding maximum cross-sensitization two days after a 15 min IMO exposure (this is possible considering the marked cross-sensitization observed after a 5 min IMO exposure in the previous experiment) and a residual cross-sensitization of 15 min IMO seven days later.

**Figure 5:**
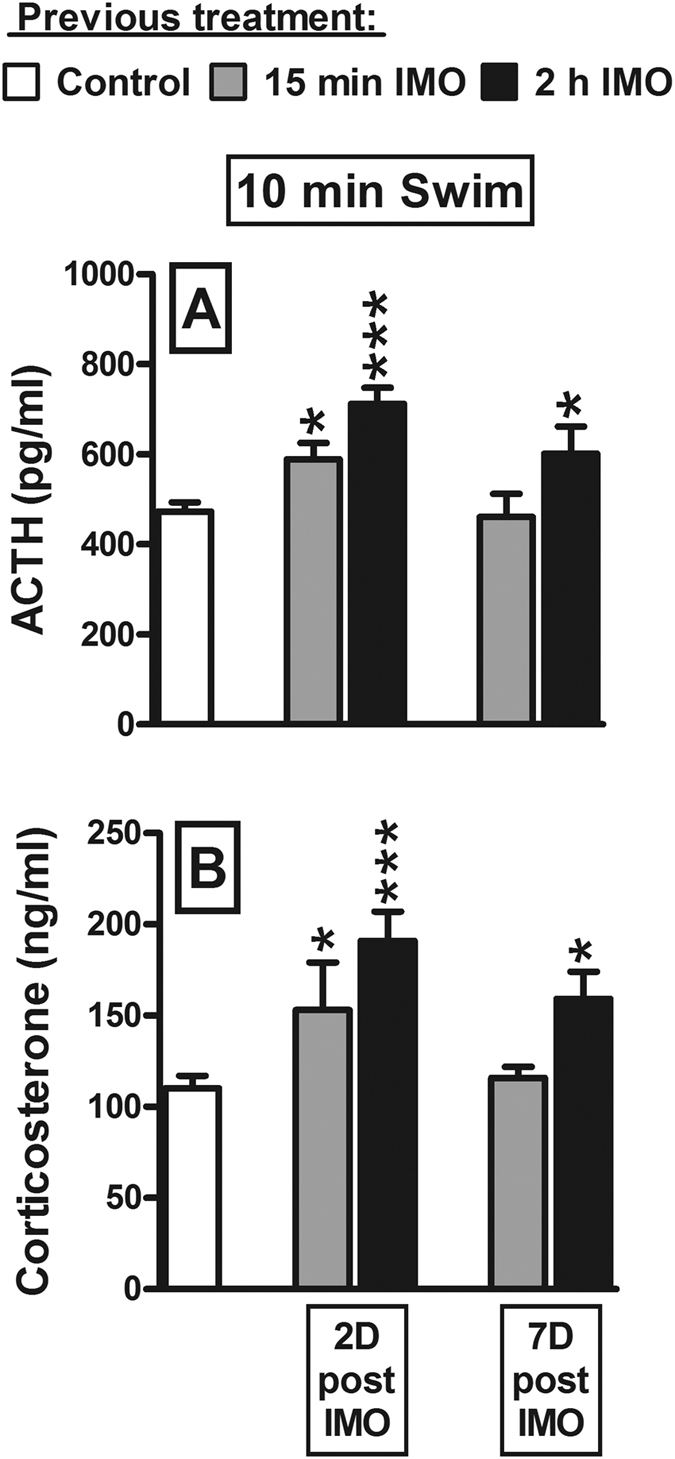
The duration of HPA cross-sensitization is dependent on the length of exposure to the triggering (IMO) stressor. The means and SEM (n = 6 per group) are shown. ACTH (**A**) and corticosterone (**B**) responses to a 10 min forced swim were studied in rats previously exposed to IMO for 15 min or 2 h, either 2 or 7 days before (2D and 7D, respectively): *p < 0.05, ***p < 0.001 versus control.

To evaluate whether prior IMO exposure actually caused HPA cross-sensitization, we analyzed the data by GzLM including only the factor prior IMO, which had a significant effect on both ACTH (χ^2^(4) = 27.7; p = 0.001) and corticosterone (χ^2^(4) = 22.0; p < 0.001). A post-hoc analysis showed that prior IMO exposure always induced cross-sensitization of the response of both hormones, with the exception of 15 min IMO exposure that did not provoke sensitization when tested 7 days later (Fig. 5A,B). These results indicate that longer prior IMO exposure translates into more persistent cross-sensitization. This conclusion was further supported by the analysis focusing on the effects of the factors IMO LENGTH and DAYS elapsed between stressors. The GzLM analysis of ACTH and corticosterone showed a significant effect of IMO LENGTH (χ^2^(1) = 9.4; p = 0.002; χ^2^(1) = 6.8; p = 0.009; respectively) and DAYS (χ^2^(1) = 7.8; p = 0.005; χ^2^(1) = 5.0; p = 0.026; respectively) but not of their interaction, indicating that the duration of prior IMO affected both the magnitude and the persistence of cross-sensitization.

## Discussion

The studies presented here define the main parameters involved in acute sensitization of the HPA response to heterotypic stressors in adult male rats. The major findings are that a brief exposure to severe stressors is enough to induce cross-sensitization, although longer exposures produce more persistent cross-sensitization. In addition, optimal cross-sensitization is observed with short exposure to the challenging stressor (i.e. 5 min) and it is masked by much longer exposure (i.e. 30 min). Finally, a lower intensity of challenging stressors favors cross-sensitization.

Although severe stressors like tail-shock (typical of the learned-helplessness paradigm) and IMO appear to consistently induce HPA cross-sensitization^17^,^18^,^19^,^20^,^21^,^29^,^30^, the specific contribution of the severity of the stressors has not previously been studied. We first studied whether HPA sensitization was dependent on the intensity of the triggering (inductive) stressor, exposing rats for 30 min to three stressors with putatively different intensities^27^,^28^: forced swim, foot-shock and IMO. Changes in body weight gain confirmed that IMO was the strongest stressor, followed by foot-shock and and then swim. We are aware that stressors can differ both in terms of intensity and also qualitatively, and that the overall physiological response to stressors that have a strong systemic component differs greatly (e.g. formalin-induced pain, insulin-induced hypoglycemia, cold, hemorrhage)^22^. However, we have systematically compared different types of stressors in which the emotional component is predominant and there are several biological markers that consistently classify them in terms of putative intensity: plasma ACTH levels, prolactin or glucose (as a surrogate of adrenaline release), and food intake or body weight gain for the most severe stressors (see ref. 23).

After exposure to the OF for 15 min on the next day, there was no sensitization of the ACTH or corticosterone response in the prior SWIM animals, whereas sensitization was evident in those exposed to prior SHOCK or IMO. A gradual effect was apparent as in SHOCK rats, sensitization was evident for the corticosterone but not the ACTH response relative to the control animals, whereas sensitization of the response of both hormones was evident in IMO rats. The lack of significant ACTH sensitization after SHOCK despite a clear corticosterone sensitization can be explained in two ways. First, sensitization might not only affect ACTH release but it may also enhance adrenal responsiveness to circulating ACTH. Second, sensitization of ACTH release could be transient (see below), mainly affecting the initial response to the stressor (i.e. first 5 min). Indeed, after a 15 min exposure ACTH levels were already declining, although sensitization is clearly observed in plasma corticosterone due to the lag time of about 15 min between the peaks of ACTH and corticosterone.

Behavior in the OF reveals a marked dissociation from the HPA response. Rats in the SWIM group did not display altered ambulatory activity in the central and peripheral part of the OF, or rearing. IMO reduced horizontal activity but not rearing, suggesting that vertical activity is more resistant to the negative impact of stress. The changes after IMO are typical of exposure to severe stressors^21^,^31^,^32^,^33^. In contrast to the relatively modest effect of IMO, foot-shock strongly inhibited both horizontal activity and rearing. The observed dissociation between HPA indexes and behavior regarding IMO and foot-shock are likely to be due to the particular properties of foot-shock in the development of cognitive generalization of fear to environments completely different from that where they experienced the foot-shock^34^,^35^,^36^,^37^. In turn, this phenomenon is associated to the acquisition of context fear conditioning^35^,^36^, a property not shared with IMO^38^. These results highlight the importance of the particular characteristics of stressors other than their intensity (defined by classical biological markers of stress) in determining long-lasting behavioral consequences. Indeed, while all severe emotional stressors might be able to induce protracted but relatively transient behavioral sensitization, only some of them might induce additional associative, more persistent behavioral effects (particularly foot-shock)^39^,^40^,^41^.

The aforementioned possibility that HPA cross-sensitization is dependent on the length of exposure to the challenging stressor is tentatively supported by the results obtained with tail-shock, whereby sensitization was clearly evident during the initial period of exposure to the novel stressor but not later^17^. However, whether the intensity of the heterotypic stressor is also important to produce sensitization has not been specifically addressed. Rats here were immobilized for 2 h the day before they were exposed for 5 min to an OF or to swim to study the contribution of the intensity of the heterotypic stressor, or to 30 min swim to determine the contribution of the duration of the heterotypic stressor. Marked IMO-induced cross-sensitization of the ACTH response was observed immediately after the 5 min exposure to the OF or to swim, yet only after the OF during the post-stress period, with no significant ACTH sensitization after 30 min swim. The pattern of the corticosterone response partially differed from that of ACTH, with clear sensitization after the 5 min OF, no sensitization after the 5 min swim and only modest sensitization after the 30 min swim. The lack of change in corticosterone in the post-swim period in the 5 min swim group is puzzling as ACTH sensitization was observed immediately after swim. However, it is possible that the effect on ACTH was so transient that it was not later reflected by a corticosterone response.

The results presented here strongly indicate that HPA cross-sensitization is dependent on the intensity of a challenging stressor, although it is much more strongly affected by the period of exposure to such stress. The critical contribution of the duration of exposure to heterotypic stressors might help explain the inconsistent results regarding the influence of prior chronic stress on the heterotypic sensitization of the HPA axis to emotional stress. A consistent response to the heterotypic stressor is not evident when assessed 30–60 min after exposure^42^,^43^,^44^,^45^,^46^,^47^, yet cross-sensitization is consistently observed when the exposure to the challenging stressor was more immediate (about 5 min)^30^,^48^,^49^. For instance, daily repeated exposure to IMO causes marked cross-sensitization of the ACTH response to a 5 min OF^30^, whereas the results differ when the same chronic stressor is coupled to a longer exposure of the challenging stressor^50^.

It is noteworthy that sensitization is particularly evident after a short exposure to the heterotypic stressors, which can be explained in different ways. First, and with the exception of social stressors, in nature emotional stressors are usually of short duration and therefore the adaptive biological consequences of HPA sensitization might have evolved to respond to such stimuli. After this initial period, sensitization could be counteracted by other events, probably related to the absence of signs of true danger. Alternatively, sensitization could actually represent an acceleration rather than an increase in the HPA response to novel stressors, offering advantages when confronted with acute stressors. For instance, faster glucocorticoid release could help improve memory consolidation related to short-lasting stressful situations.

While acute severe stressors consistently induce HPA cross-sensitization, the temporal threshold of these stressors to induce sensitization is unknown. We immobilized rats for 5 min, 30 min or 4 h and studied sensitization of the HPA response to a short OF exposure on the following day. Surprisingly, HPA sensitization was of similar magnitude after the 5 and 30 min IMO, with only a small increase after 4 h IMO. These results not only demonstrate that HPA sensitization can be produced by a brief exposure to traumatic stressors but also, that negative glucocorticoid feedback is probably not operative under stress as the prolonged activation of the HPA axis caused by 4 h IMO did not impair the HPA response to additional stress on the next day. The lack of effect of stress-induced glucocorticoid release on negative feedback is consistent with the hypothesis that exposure to stress facilitates HPA responsiveness, thereby overcoming glucocorticoid negative feedback^16^. This facilitation may also explain the normal response to stress in the 4 h IMO group, despite the elevated resting levels of corticosterone prior to exposure to the challenging stressor. To directly demonstrate that exogenous administration of glucocorticoids is far from mimicking the complex effects of stress on the activity of the HPA axis, we introduced a group of rats given exogenous corticosterone to simulate the release caused by 4 h IMO. Although plasma corticosterone levels were somewhat higher after IMO than after corticosterone administration at 1 h, these levels were similar at 4 h and somewhat lower at R45. Consequently, the AUCs were similar, even though exposure to 4 h IMO caused sensitization whereas corticosterone administration completely blocked the ACTH response to the OF, indicating that a strong negative feedback was still present at this time. Although the impact of glucocorticoid negative feedback on acute stress-induced HPA responsiveness is not usually tested in animals on the day after a single administration of glucocorticoids, there is some evidence for similar long-lasting effects of cortisol administration^51^. As such, it appears that IMO-induced facilitation of the HPA axis could fully overcome glucocorticoid negative feedback. Alternatively, glucocorticoid released during a stressful situation may not trigger negative feedback because such a mechanism is not operative under stress. This second possibility is compatible with our earlier findings that administration of the glucocorticoid receptor (GR) antagonist mifepristone prior to IMO did not further enhance IMO-induced HPA cross-sensitization^21^.

Although a brief exposure to IMO is enough to induce HPA cross-sensitization, the persistence of the sensitized state could be dependent on the length of initial exposure to IMO. This possibility was tested in suboptimal conditions of the challenging stressor to better discriminate between the effects of two IMO periods, both of which elicited ACTH and corticosterone cross-sensitization, although the effect was greater after longer IMO. Seven days after IMO, significant sensitization was restricted to this latter IMO group, albeit of lower magnitude than that observed on the following day. Hence, HPA cross-sensitization is longer lasting after a more prolonged exposure to the inductive stressor.

The precise mechanisms involved in acute stress-induced cross-sensitization of the HPA axis remain largely unknown^7^, although this phenomenon appears to be located at the level of the PVN, or above rather than at the level of the anterior pituitary^19^. Interestingly, pharmacological activation of the HPA axis with metyrapone does not induce HPA cross-sensitization^21^, despite a marked activation of CRH gene expression and ACTH release^52^,^53^. Moreover, cross-sensitization does not require activation of CRH-1 receptors^21^. Hence, HPA cross-sensitization might be specifically associated with strong emotional activation caused by severe stressors. Since cross-sensitization was observed after a short exposure to IMO, severity rather than duration would appear to determine sensitization, which is important when considering the possible consequences of trauma in humans. Unfortunately, to the best of our knowledge there are no studies into HPA responsiveness to stress on the days following exposure to traumatic situations in humans.

It is unclear to what extent the data on chronic stress-induced cross-sensitization, and particularly that related to the facilitation of the HPA axis, can be extrapolated to acute stress. Moreover, the inconsistency of chronic stress-induced facilitation of the response to heterotypic stressors^54^,^55^,^56^ represents an additional problem. Chronic stress is likely to be affected by the same factors as those described for acute stress-induced HPA facilitation, for instance the duration of the challenging stressor or the type and/or intensity of the challenging stressor^45^. Moreover, models of chronic stress that include severe systemic stressors are more likely to detect cross-sensitization, as is the case of hypertonic saline^57^, chronic cold exposure^58^,^59^ (although see ref. 60) and some models of chronic unpredictable stress^61^,^62^. Importantly, the systemic nature of the stressor appears to be critical for sensitization of the central noradrenergic system (see ref. 7). Localizing brain areas where a prior history of chronic stress could induce enhanced neuronal activation (as evaluated by c-fos and other immediate early genes) has given inconsistent results that are difficult to explain^43^,^46^,^58^,^61^,^63^,^64^.

There is evidence that the posterior region of the paraventricular thalamic nucleus (pPVTh) plays a role in chronic stress-induced cross-sensitization, while not influencing the HPA response to acute stressors in stress-naïve rats^58^. Some neuropeptides that act at the pPVTh appear to play important roles in cross-sensitization. Orexin inputs to the nucleus are important for the induction but not for the expression of facilitation^65^, whereas the expression of the phenomenon is restrained by cholecystokinin acting through CCK-B receptors^59^. Unfortunately, the inputs to the pPVth in this process and the pathways linking the pPVTh to the PVN remain unclear. Nevertheless, HPA cross-sensitization probably does not involve impaired fast or delayed negative glucocorticoid feedback^44^,^62^. Although the non-hypothalamic brain CRH pathways appear to influence the induction or expression of cross-sensitization between chronic stress and addictive drugs, as well as some effects of chronic stress on brain noradrenergic activity^66^,^67^,^68^,^69^,^70^, there is no evidence of a critical role of CRH in chronic-stress induced cross-sensitization of the HPA axis.

In conclusion, the results presented here demonstrate that HPA sensitization is critically dependent on the characteristics of both the triggering (inductive) and the challenging stressor. Specifically, the intensity of the inductive stressor is more critical than its duration, although longer exposures to stressors can induce more persistent sensitization. Rather than its intensity, the length of exposure to the triggering stressor is most important to provoke sensitization, which may explain the controversial results in the literature and particularly those regarding chronic stress-induced HPA sensitization.

## Materials and Methods

### Animals and general procedures

Male Sprague-Dawley rats from the Animal Facility of the Universitat Autònoma de Barcelona (UAB) were used in these studies. The rats were 2–3 months old at the beginning of the experiments and they were housed in pairs under standard conditions: temperature (21 ± 1 °C), 12 h light cycle (lights on 07:00–19:00 h), and food and water *ad libitum*. All animals were habituated to handling (at least three times) and blood-sampling by the tail-nick procedure^21^ (once) prior to the experiments, which were always carried out in the morning. Cage-mates were always tested simultaneously. The experimental protocol was approved by the Ethics Committee at the Universitat Autònoma de Barcelona and the Generalitat de Catalunya, and it was carried out in accordance to the European Council Directive (2010/63/UE) and Spanish legislation (RD 53/2013).

### Stressors

Forced swim: rats were placed individually in transparent cylindrical plastic tanks (height = 40 cm, internal diameter = 19 cm) containing water (36 °C) to a level of 24 cm^71^.

Foot-shock: rats were placed in shock chambers made of clear Plexiglas boxes (57 × 41 × 70 cm) with a removable metal grid on the floor composed of 44 stainless steel rods (0.4 cm diameter) that were spaced 1.5 cm apart, center-to-center^38^, and they received one 3 s, 1.0 mA, foot-shock each min.

Immobilization (IMO): the four limbs of the rats were taped to metal mounts attached to a board, as described previously^21^.

Open-field test (OF): The OF was a plastic gray rectangular box (56 × 36.5 × 31 cm) open at the top, where each animal was initially placed facing a corner. Exposure to novel environments is a mild stress that allows behavioral assessment. Between animals, the apparatus was cleaned carefully with tap water containing ethanol (5% v/v). OF behavior was recorded with a video camera (Sony SSC-M388 CE, BW) situated 150 cm above the center of the cage. The box was divided into 12 areas (10 of them in contact with the walls and 2 in the center of the box) and the number of areas crossed (peripheral and central) was calculated manually^21^.

### Radioimmunoassays

Plasma ACTH and corticosterone levels were analyzed using radioimmunoassays as described previously^72^. All samples to be statistically compared were processed in the same assay to avoid inter-assay variability.

### Statistical analysis

The statistical package for social science (SPSS, version 23 for Windows) software was used for the analysis. The AUCs (calculated using Graph-Pad Prism 5.0) of the hormonal data were analyzed using a 2-tailed Student’s *t*-test. For the rest of the comparisons, statistical analyses were performed using the GzLM when only between-subjects factors were included^73^ and the GEE model when within-subjects factors were present in the analysis^74^. The between-subjects factor was either the treatment on day 1 or day 2, and the sampling time was the within-subjects factor (when more than one blood sample was obtained). These models are a more flexible statistical tool than the standard general linear model (GLM) because several types of distribution and different covariance structures of the repeated measures data could be chosen. In addition, the GzLM does not require homogeneity of variances and it admits missing values without removing all the subject data. The significance of the effects was determined with the Wald chi-square statistic (χ^2^). After the main analysis, appropriate pair-wise comparisons were carried out using the sequential Bonferroni corrections. The criterion for significance was set at p < 0.05 for all tests.

## Additional Information

**How to cite this article**: Belda, X. *et al.* Critical features of acute stress-induced cross-sensitization identified through the hypothalamic-pituitary-adrenal axis output. *Sci. Rep.* **6**, 31244; doi: 10.1038/srep31244 (2016).

## Supplementary Material

Supplementary Information

## Figures and Tables

**Table 1:** Scheme of the triggering and challenging stressors used in the experimental procedures.

	Triggering Stressor(s)	Challenging Stressor(s)
Exp. 1	Forced Swim (30 min)Foot-shocks (30 min)IMO (30 min)	Open Field (15 min)
Exp. 2	IMO (2 h)	Open Field (5 min)Forced Swim (5 min)Forced Swim (30 min)
Exp. 3	IMO (5 min)IMO (30 min)IMO (4 h)Corticosterone	Open Field (5 min)
Exp. 4	IMO (15 min)IMO (2 h)	Forced Swim (10 min; 2 or 7 days later)
