# Supplementary material for: Critical features of acute stress-induced cross-sensitization identified through the hypothalamic-pituitary-adrenal axis output
Source: Sci Rep. 2016 Aug 11;6:31244. doi: 10.1038/srep31244 (PMC4980629; doi:10.1038/srep31244)
Supplement: Supplementary Information [file srep31244-s1.doc]

**Critical features of acute stress-induced cross-sensitization identified through the hypothalamic-pituitary-adrenal axis output**

**Xavier Belda, Roser Nadal & Antonio Armario**

**Supplementary Table 1. Plasma levels of ACTH and corticosterone (mean ± SEM) in rats remained undisturbed (CONTROL, n=10) or subjected to 5 or 30 min of IMO (n=8 per group).** Blood samples were collected in CONTROL and IMO groups following the same schedule: just after IMO (END) and again 45 min later (R45).

| **GROUP** | **ACTH (pg/ml)** | | **Corticosterone (ng/ml)** | |
| --- | --- | --- | --- | --- |
|  | **END** | **R45** | **END** | **R45** |
| **CONTROL** | 82 ± 5 | 91 ± 4 | 34 ± 11 | 60 ± 15 |
| **5 min IMO** | 726 ± 48 | 224 ± 28 | 115 ± 10 | 488 ± 61 |
| **30 min IMO** | 1353 ± 64 | 318 ± 31 | 635 ± 52 | 509 ± 34 |
